# Animal models of chronic obstructive pulmonary disease: a systematic review

**DOI:** 10.3389/fmed.2024.1474870

**Published:** 2024-10-24

**Authors:** Tiantian Feng, Juan Cao, Xiaoting Ma, Xinhua Wang, Xiaolong Guo, Na Yan, Chunling Fan, Shisan Bao, Jingchun Fan

**Affiliations:** ^1^School of Public Health, Centre for Evidence-Based Medicine, Gansu University of Chinese Medicine, Lanzhou, China; ^2^Department of Public Health, Affiliated Hospital of Gansu University of Chinese Medicine, Lanzhou, China; ^3^School of Nursing, Gansu University of Chinese Medicine, Lanzhou, China; ^4^Department of Clinical Pharmacy, Gansu Provincial Cancer Hospital, Lanzhou, China

**Keywords:** COPD, animal models, modelling method, review–systematic, smoke + LPS

## Abstract

**Objective:**

Experimental animal models have been used for decades to study the development and progression of chronic obstructive pulmonary disease (COPD). However, there is a lack of methods for constructing animal models of COPD for optimal modelling. This systematic literature review (SLR) aimed to assess the various methods used to establish COPD animal models, highlight their advantages and limitations, and explore more optimized approaches for establishing such models.

**Methods:**

A systematic search was performed in four English databases (PubMed, Embase, Web of Science, and the Cochrane Library) and four Chinese databases (Chinese Biomedical Literature Database, China National Knowledge Infrastructure, China Science and Technology Journal Database, and Wanfang Database). Of the 8,015 retrieved full-text manuscripts, 453 were selected.

**Results:**

Smoking (*n* = 140), smoking combined with lipopolysaccharide (LPS) (*n* = 275), smoking combined with protease drip (PPE) (*n* = 10), smoking combined with bacteria (*n* = 23), and smoking combined with particulate matter (PM2.5) (*n* = 5) were the most used methods for establishing animal models of COPD. Rats and mice were the most frequently selected experimental animals, with male animals accounting for 79.47% of the total. A total of 92.49 and 29.14% of the articles reviewed considered lung pathology of experimental animals only and lung pathology and lung function tests, respectively.

**Conclusion:**

Our review suggests that the best way to establish an animal model of COPD is to combine smoking with LPS. Although findings from animal models of COPD cannot be directly extrapolated to human COPD, they could provide useful tools for further investigation into human COPD disease.

**Systematic review registration:**

https://www.crd.york.ac.uk/prospero/display_record.php?ID=CRD42023407555, Identifier PROSPERO CRD42023407555.

## Introduction

Chronic obstructive pulmonary disease (COPD) is a respiratory disease characterized by a complex pathogenesis, prolonged course, and persistent airflow limitation (1–3). It is the third leading cause of death worldwide and has become an increasingly important public health problem (4). The disease has high morbidity and mortality rates, and severe cases may experience problems, such as a progressive decline in lung function, respiratory failure, and pulmonary heart disease (5, 6). COPD is also characterized by high incidence, high disability rates, long duration, and significant comorbidities, which seriously affect the quality of life of patients and impose a substantial economic burden on both patients and society (7, 8). The incidence of COPD in Southwest China has increased significantly, resulting in higher costs of COPD inpatient and outpatient care (9).

The etiology of COPD is complex, primarily due to infection or air pollution, although in some cases, the cause is unclear (10, 11). The precise underlying mechanism of COPD is not fully understood, which has led to significant limitations in its treatment (12, 13). The risk factors for developing COPD are diverse and can be summarized as individual susceptibility and environmental factors (14). Individual susceptibility includes genetic factors (15), age and sex (16, 17), lung growth and development (18), asthma and airway hyperresponsiveness (19), and low body mass index (20). Environmental factors include cigarette smoke (21), air pollution (22), and bacterial (23) or viral (24) infections, which can cause varying degrees of respiratory inflammation, potentially leading to lung infections and ultimately COPD (25).

To gain a deeper understanding of the disease, many researchers chose to conduct COPD experiments in animals, which serve as “human stand-ins” to simulate various diseases (26). Establishing an animal model of COPD is thus a key approach for exploring the underlying mechanism for treatment (27). The complexity of COPD’s etiology necessitates a variety of methods for establishing animal models of COPD (28). Current methods with high success rates include smoking alone, smoking combined with lipopolysaccharide (LPS) (29), smoking combined with protease drip (PPE) (30), smoking combined with bacterial drip, and smoking combined with particulate matter (PM2.5) drip or intermittent hypoxia (31, 32). In addition, successful animal models of COPD can be established by the following methods: smoking combined with LPS and protease (33), smoking combined with SO_2_ (34), smoking combined with LPS and cold wind stimulation (35), smoking combined with LPS and ozone (36), bleomycin administration (37), genetic manipulation (38), cigarette smoke extract (CSE) (39), fluorescein isothiocyanate administration (40), and silica administration (41).

Although there are various approaches through which COPD animal models can be established, the modelling process is challenging, and no single *in vivo* model can fully replicate the pathological features of human COPD, especially in the later stages of the disease (25, 42).

## Methods

A systematic literature review (SLR) based on a protocol (43) was conducted, utilizing four English databases (PubMed, EMBASE, Web of Science, and the Cochrane Library) and four Chinese databases (Chinese Biomedical Literature Database, China National Knowledge Infrastructure, China Science and Technology Journal Database, and Wanfang Database). The search strategy for PubMed is provided (Table 1). Table 1 shows the search process for one smoking-induced COPD model on PubMed. However, four additional models (smoking combined with LPS, smoking combined with bacteria, smoking combined with PM2.5 particulates, and smoking combined with protease) were also searched on PubMed. Due to space limitations, the additional four tables are not shown. The search was conducted in December 2023. In addition to the electronic database searches, manual searches were also conducted to capture data from recent studies not yet published.

**Table 1:** Search strategy (PubMed).

#1	Search “Pulmonary Disease, Chronic Obstructive” [Mesh]
#2	Search: (((((Chronic Obstructive Lung Disease [Title/Abstract]) OR (Chronic Obstructive Airway Disease [Title/Abstract])) OR (Chronic Obstructive Pulmonary Disease [Title/Abstract])) OR (Chronic Air Flow Obstructions [Title/Abstract)) OR (Chronic Airflow Obstruction [Title/Abstract])) OR (COPD [Title/Abstract])
#3	Search: “Tobacco Products”[Mesh]
#4	Search:(((((Cigarettes [Title/Abstract]) OR (Cigarette [Title/Abstract])) OR (Cigars [Title/Abstract])) OR (Cigar [Title/Abstract])) OR (Cigarillos [Title/Abstract])) OR (Cigarillo [Title/Abstract])
#5	Search: “Models, Animal” [Mesh]
#6	Search: (((((Animal Model [Title/Abstract]) OR (Animal Models [Title/Abstract)) OR (Laboratory Animal Model [Title/Abstract)) OR (Laboratory Animal Models [Title/Abstract)) OR (Experimental Animal Model [Title/Abstract])) OR (Experimental Animal Models [Title/Abstract])
#7	Search:#1 OR #2
#8	Search: #3 OR #4
#9	Search: #5 OR #6
#10	Search: #7 AND #8 AND #9

### Eligibility criteria and study selection

The study question of this systematic literature review (SLR) was specified using the population, intervention, comparison, outcomes, and study (PICOS) (design framework). Articles were retrieved using a set of search terms specific to animal models of chronic obstructive pulmonary disease, and they were then further filtered to find those that met the coding criteria. Eligible articles were those that met the following criteria:

1. Type of participants: All animals with successfully established COPD models were included, regardless of species, sex, or age. Animals without COPD (such as those with other diseases or health issues) were excluded.
2. Type of design: There were no restrictions on the types of studies. However, case reports, reviews, conference abstracts, mechanism discussions, and empirical summaries were excluded.
3. Type of interventions: Studies that created animal models of COPD using the following five methods were included: smoking, smoking combined with LPS, smoking combined with bacteria, smoking combined with PM2.5, and smoking combined with protease. Other types of COPD animal modelling methods not mentioned in the inclusion criteria were excluded.
4. Type of comparators: Control or comparison conditions included animals that did not have an established COPD model, such as those living under normal conditions without any intervention. Studies on the establishment of COPD animal models using other methods were excluded.
5. Type of outcomes: The main outcome indicators were the pathological changes in the lungs of COPD animal models, as well as lung function indicators and the number of inflammatory cells produced.
6. Other criteria: There were no limitations on the year of publication, language, or publication status. However, articles with repeated publications, incomplete data, or unclear outcome indicators were excluded.

### Literature screening and data extraction

All authors of this study had experience in completing systematic reviews. Each citation retrieved was reviewed by two independent reviewers against the eligibility criteria. We imported the retrieved articles into EndNote 20.2.1 (Build 15,749, Clarivate), first performing automatic rearrangement through the software and then manual rearrangement based on basic information, such as article title and author. After excluding duplicates, the two reviewers assessed the titles and abstracts according to the inclusion and exclusion criteria. The articles that could not be identified were evaluated through a full-text reading. During this process, any disagreements between the reviewers were resolved through discussion and consultation with a third reviewer.

### Quality assessment

Two researchers independently assessed each study, and a consensus discussion was used to resolve any disagreements. During the study, we evaluated the quality of the experimental animals in strict accordance with the seven items listed in the Stroke Therapy Academic Industry Roundtable (STAIR) checklist. The STAIR checklist was used to evaluate the quality of the animal experiments, and its seven items served as the criteria for assessing this quality:

1. Sample size calculation.
2. Inclusion and exclusion criteria.
3. Random sequence generation.
4. Concealment of grouping protocol for laboratory animals.
5. Reasons for excluding animals from the analysis.
6. Blind evaluation of outcomes.
7. Declaration of potential conflicts of interest and research funding.

### Statistics

Descriptive statistics were used to characterize the included studies. Figures depicting these descriptive statistics were created using GraphPad Prism 8.0.2 for Mac OS X (GraphPad Software, San Diego, CA, United States).

## Results

### Literature search and screening

**Figure 1:**
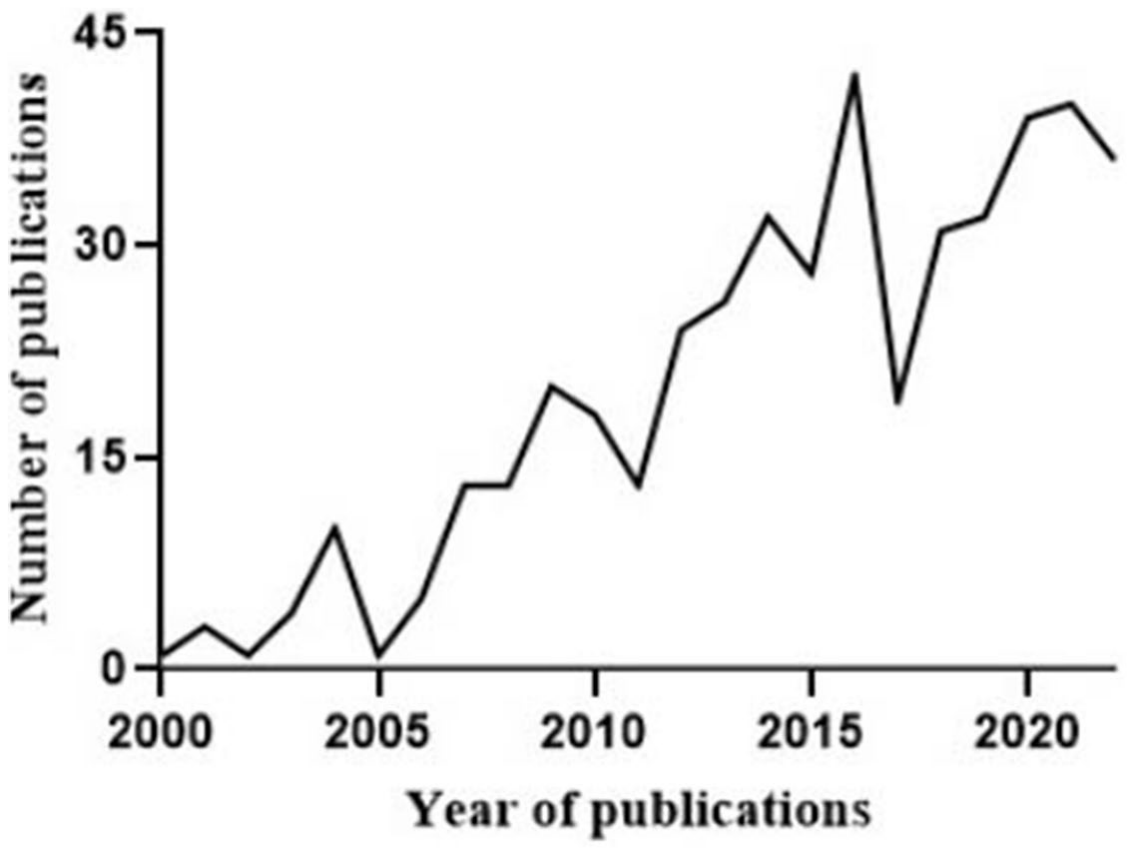
The flow diagram for study selection.

A total of 8,015 citations were identified through the electronic database search. Duplicates were identified and compared based on an exact match for author, year, title, and abstract. After removing the duplicates, 2,088 unique citations were obtained and screened. Of these, 1,635 full articles were excluded: 626 publications were excluded because they did not contain appropriate data, 407 documents were excluded because the investigators presented methods of modelling without model evaluation, 82 documents were excluded because there was no full text available, and 520 documents were excluded due to duplication. After screening the titles, abstracts, and full texts according to the predetermined selection criteria, a total of 453 articles that met our inclusion criteria were included (Figure 1). The details of the 453 included articles are provided in the table of included studies (Appendix 1). A summary is described below.

### Characteristics and classification of the included studies

**Figure 2:**
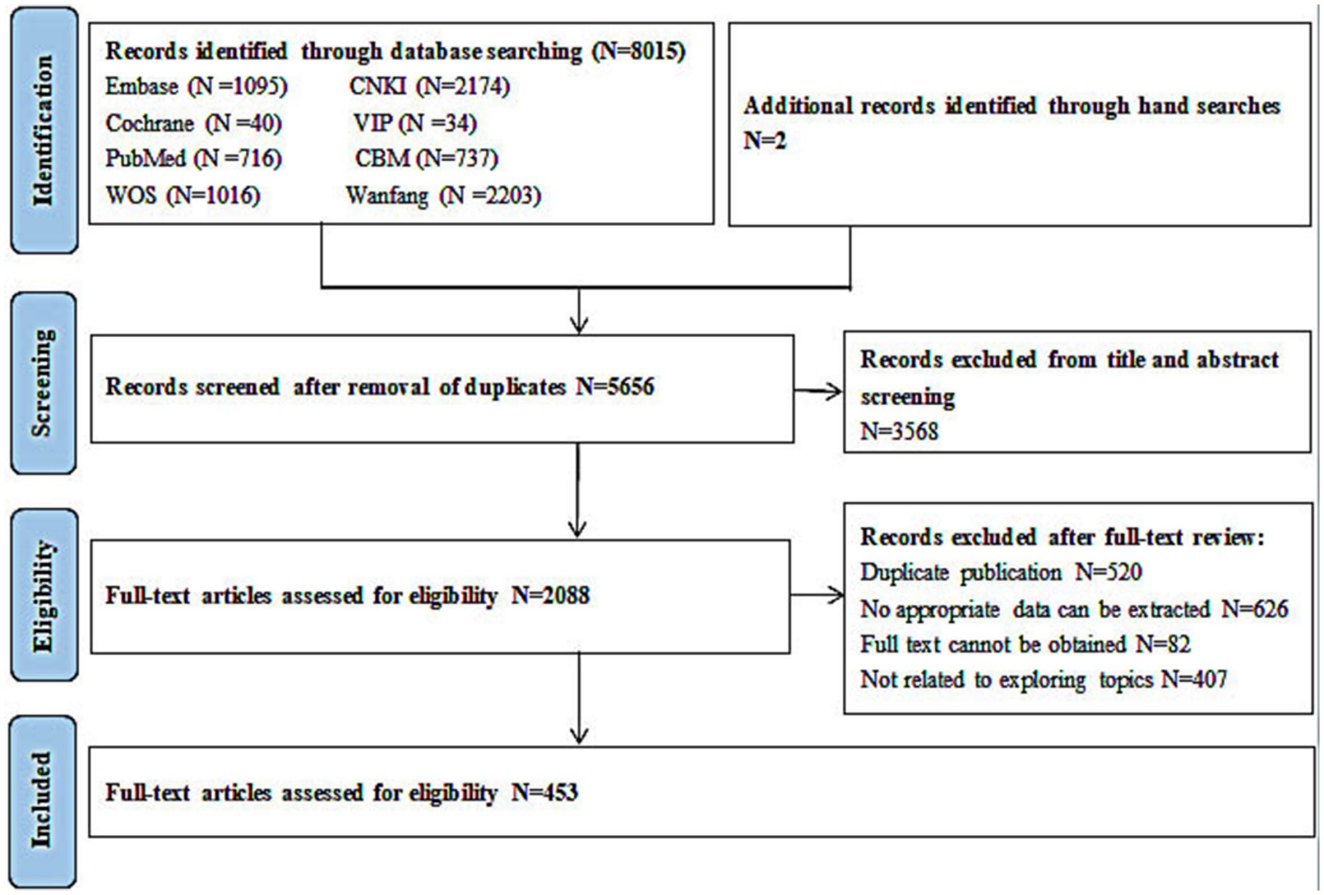
The number of the included studies sorted by the year of publication.

The publication dates of the literature included in this systematic review ranged mainly from 2009 to 2022, as shown in Figure 2. A total of eight different animals were involved in establishing animal models of COPD in the included studies. The most commonly used animal was the rat, which was used in 389 studies (85.9%), followed by the mouse, which was used in 53 studies (11.7%). The guinea pig, hamster, cat, fruit fly, rabbit, and ferret were modelled in 4 (0.9%), 3 (0.7%), 1 (0.2%), 1 (0.2%), 1 (0.2%), and 1 (0.2%) studies, respectively. Young mice and rats with a median age of 8 weeks were predominantly used in the studies included in this review, although the ages of the mice and rats ranged from 3 to 54 weeks and 5 to 14 weeks, respectively. The animal characteristics are presented in Table 2.

**Table 2:** Frequency distribution of experimental species in a model of COPD.

Species	Frequency	Percentage	Body weight	Sex	Advantage	Disadvantage
Rats	389	85.9	Mainly (200 ± 20) g	Male (321/389)	Large body size, easy to measure lung function. The cost effective in feeding and drug usage.	Rats are resistant to the development of COPD. Rats had no goblet cells and had significantly fewer mucosal glands than large animals.
Mice	53	11.7	Mainly (20 ± 2) g	Male (31/53)	Fast breeding. Easy manoeuvre. Cost effective.	Difficult to obtain alveolar lavage fluid due to small airway. Smoking pattern is different from human due to different anatomy from human.
Guinea pig	4	0.9	Mainly (370 ± 2) g	Male (3/4)	Similar inflammatory responses with human. Similar histological structure with human in airway.	Most of the lung injury is axonal reflex. Relatively cost expensive. Difficult to perform airway manoeuvre.
Hamsters	3	0.7	Mainly (100 ± 20) g	Male (3/3)	Have abundant non-ciliated bronchiole cells and goblet cells, closer to human in anatomy, physiology, genetics and biochemistry.	Difficult to manoeuvre due to more aggressive.
Rabbits	1	0.2	Mainly (1,500 ± 200) g	Female (1/1)	Large in size and long-life span for long-term studies.	Susceptible to bacterial and viral infections during sterility tests.
Cat	1	0.2	Mainly (2000 ± 500) g	Male (1/1)	Enable parallel study *in vivo* and *in vitro.* Pulmonary inflammation is similar to humans.	Cost expensive.
Fruit fly	1	0.2	Not described		Its respiratory system is similar to the mammalian. Early damage to airway can be detected.	Difficult in handling due to small in size.
Ferret	1	0.2	Not described		The model relates to the complex clinical features of human COPD. Could be used for long-term research.	Costs is substantially high, as well as difficult in handling.

The modelling methods used in different studies varied, and this article incorporated the most used modelling methods up to this point. The commonly used methods for COPD animal models were smoking (*n* = 140), smoking combined with LPS (*n* = 275), smoking combined with protease infusion (10 articles), smoking combined with bacterial respiratory infection (*n* = 23), and smoking combined with particulate matter (PM2.5) (*n* = 5). The findings from the literature review showed that smoking combined with LPS was the most common method (60.7%) used to establish animal models of COPD. At the same time, the data showed that the use of cigarettes in combination with other methods required less time to establish COPD animal models and produced more pronounced pathological changes in the lungs. The pros and cons of the different methods used for establishing animal models of COPD and the frequency of their use are shown in Table 3.

**Table 3:** Classification and frequency of use of COPD models.

Method	Frequency	Percentage	Advantage	Disadvantage
Smoke exposure	140	30.9	Convenient operation and low cost. Stable animal models can be obtained after sufficient smoking time.	The length of time required to establish the model. High instability during smoking. Prone to adverse conditions such as death.
Smoke exposure combined with lipopolysaccharide	275	60.7	Reducing the time to cure COPD. Improve stability of animal models. The success rate of establishing model is high.	The preparation method is influenced by many factors. The timing and dosage of LPS administration varies.
Smoke exposure combined with protease infusion	10	2.2	Reducing the time to cure COPD. The degree of the pathology of COPD can be controlled by adjusting the amount of the protease.	Operation is difficult. Subsequent infections may occur.
Smoke exposure combined with bacterial infection	23	5.1	The method is easy to operate. Less trauma to the animal during model establishment.	The volume of bacterial suspensions inhaled by animals is difficult to quantify accurately.
Smoke exposure combined with air pollutants (PM2.5)	5	1.1	The method is easy to operate. Less trauma to the animal during model establishment. The animal models formed are more stable.	Longer model build time and less effective. Pollutant concentrations are difficult to control.

Most studies were performed in experimental animals of the male sex. Among the studies performed in rats, mice, and guinea pigs, 321 studies (82.5%) used male rats, 31 studies (58.5%) used male mice, and 3 studies (75.0%) used male guinea pigs, respectively. Male animals were also chosen for the hamster and cat studies, while female animals were chosen for the rabbit study. The number of articles that used either female animals or a 50/50 split between male and female animals in their studies was very small. Of the included literature, 92.5% described changes in the lung histopathology and inflammatory factors in the animals after modelling, and 29.1% of the studies included both lung function tests and observations of lung histopathology and inflammatory factors in the animal models of COPD.

### Quality assessment

Among the 453 included animal studies, most of the studies met two of the STAIR checklist requirements: calculation of sample size and generation of random sequences (62.7%). A small proportion of the studies met three of the checklist items: calculation of sample size, generation of random sequences, and reporting of reasons for excluding animals from analysis or reporting of potential conflicts of interest and research funding (28.5%). Few articles met four items in the checklist (7.3%) (Table 4).

**Table 4:** The quality statistics of 453 articles were included.

List regulations	Number (*N*, %)
random sequence generation	5, 1.1%
Calculation of sample size	1, 0.22%
Exclusion cause analysis	0
Declare conflict of interest	1, 0.22%
random sequence generation + Calculation of sample size	284, 62.7%
random sequence generation + Calculation of sample size + Exclusion cause analysis	54, 11.9%
random sequence generation + Calculation of sample size + Declare conflict of interest	75, 16.6%
random sequence generation + Calculation of sample size + Exclusion cause analysis + Declare conflict of interest	33, 7.3%

## Discussion

Animal models of disease could successfully and accurately recapitulate many aspects of human disease to provide the conditions for an in-depth study of various interventions and pathophysiology (42). This systematic review analyzed 453 studies using different types of animal models. There was a correlation between the method of establishing the COPD model and the choice of experimental animals and the date of publication of the included articles. The majority of the 453 articles included in this study were published between 2009 and 2023, providing a reasonable representation of modelling COPD in recent years. Previous studies suggested that the most common method of establishing COPD animal models was smoking and the most common experimental animals were mice, but this study found that rats were the most common animals used to establish COPD models and that the most common method of modelling COPD animals has changed from smoking alone to smoking combined with LPS. This may be due to the differences in the number of articles included and the timing of publication.

Animal species currently available for modelling COPD include mice, rats, guinea pigs (44–46), fruit flies (47, 48), dogs (49), pigs (50), ferrets (51–53), and monkeys (54), among which the most widely used are rodents (55). The genetic similarity between rodents and humans is 90% (56), and the pathogenesis and pathological damage are highly correlated with those in humans. Rodents are chosen for their short reproductive cycle, low feeding costs, low respiratory filtration rate during modelling, and ease of establishing stable animal models of COPD. Medium-sized animals, such as rabbits, cats, pigs, dogs, monkeys, horses, and sheep have also been used in the development of animal models of COPD (49, 54, 57–61). The medium-sized animal is more similar to humans in terms of anatomy, genetics and physiology, and its long lifespan allows it to be used for long-term studies but at a higher experimental cost. The physiology and genome of non-human primates (NHPs) are very similar to those of humans, but they are less commonly used due to the high cost and ethical implications associated with them (62). In contrast, the fruit fly COPD model is primarily used to screen for genes associated with human COPD (63). Thus, the selection of animal models is heavily dependent on the personal expertise of the researchers, the availability of the laboratory facility, and the financial situation of the researchers. It is evident that a large-sized animal model would be more suitable for the development of COPD, which can closely mimic human conditions for medical research. On the other hand, small-sized animal models, particularly with genetical manipulation, offer more versatile approaches for the determination of COPD at different time points and/or dosages to investigate the underlying mechanism.

Currently, mice are used in many studies to establish animal models of COPD. The advantage of using mice is that they offer a cost-effective option with proper sex, genetic, and environmental controls. At the same time, we also found that the pathological changes in C57/BL6 mice were weaker than those in other species after the same modelling period. This indicates that C57/BL6 mice are more resistant to smoke-induced changes.

### COPD animal modelling methods

A variety of methods have been used to establish animal models of COPD over the past few decades, but there is no perfect modelling method that allows animal models to accurately reproduce the pathological and physiological changes in human COPD. Each model has its own advantages and disadvantages.

The elastase model of COPD has been commonly used for research. Recently, smoking alone has increasingly been used to establish COPD animal models; however, this method also has certain limitations (64). With the further deepening of the understanding of COPD in recent years, to shorten the modelling time and, at the same time, to present a better modelling effect, a combined method is now often used to establish COPD animal models.

When the smoke exposure method is used to establish COPD models, the duration, frequency, number, and type of exposures vary, as do the lung pathological changes in the animal models (65). There are two methods of smoke exposure: whole-body smoke exposure and smoke exposure through the mouth or nasal passages only. Different smoking methods make the smoke concentration inhaled by experimental animals different, which is emphasized as a potential source of phenotypic variation in mice (65, 66). The literature shows that animal models of COPD established by nasal exposure alone produce phenotypic changes that are more similar to those seen in humans (67). Animal models of COPD established by short-term smoking (≤ 7d) can be used to characterize the disease in the early stage, but they have the disadvantage of the histopathological changes in the animal models being not stable (68–70). Therefore, animal models of COPD induced by long-term smoking (≥ 3 months) are more useful for studying the pathological mechanisms of COPD in the stable phase (71, 72). This leads to the conclusion that the selection of the stimuli and/or time is based on the establishment of the animal model as acute and/or chronic.

In addition, the combined modelling method of smoking combined with LPS or PPE is more preferred by researchers. Smoking combined with LPS to establish COPD animal models is the most commonly used method (73). Long-term exposure to LPS causes alterations in the lungs of experimental mice. Inflammatory factors increase rapidly, causing typical symptoms of COPD, such as decreased lung function, emphysema, airway hyper responsiveness, inflammatory cell infiltration, excessive mucus secretion, and pulmonary fibrosis, which in turn lead to increased destruction of the extracellular matrix, thus triggering COPD (74–80). Combined stimuli provide the benefit of combined stimulation in the development of COPD animal models. The establishment of COPD animal models by smoking combined with PPE infusion is more suitable for the preliminary evaluation of alveolar injury mechanisms (81, 82). However, care must be taken to keep the dose of protease within a reasonable range for application (77, 83, 84). Such findings indicate that researchers should select different biological stimuli based on the purpose of the clinical severity, pathological damage, or pathogenesis of COPD. Depending on the purpose of the study, smoke combined with bacteria or particulate matter (PM2.5) can also be used to establish animal models of COPD (85–87). In addition, animal models of acute exacerbation of COPD (AECOPD) can be established. However, the bacteria can damage other organs in the animals, which could affect the results of subsequent studies (88). Thus, such models offer the opportunity to determine the pathogenesis of bacterial and/or pollutants-induced COPD during an acute attack, especially by allowing for the control of the timing and dosage.

### Outcome measurement

The current systematic evaluation found that most researchers used male animals to establish animal models of COPD; the reason for the choice of male animals may be twofold: first, male animals do not have the potential to interfere with the experimental kinetic cycle and second, it helps avoid the effects of female estrogen secretion on the modelling (89). In 1999, the Committee on Understanding the Biology of Gender and Sex Differences, established by the Institute of Medicine (IOM) of the National Academy of Sciences, US proposed that (90) “Sex matters, because women have 4 levels of ovarian cycles, leading to increased experimental costs” (91). Therefore, it is recommended that male animals be used for initial studies of drug efficacy and that both sexes be used for more detailed studies of the characteristics and mechanisms of drug action.

Histopathology is mainly used to determine the extent of pathological changes in the lungs and provide information on the inflammatory cell infiltration and lung interval size. A simultaneous observation of the lung histopathology, lung function test, and inflammatory factor infiltration was performed in 29.1% of the studies on animal models of COPD. The use of a variety of outcome tests helps researchers look at the likelihood of changes in the outcome indicators from different perspectives. This information can also help researchers evaluate the pathophysiological mechanisms of COPD. In addition, data can be obtained at the immunopathological level (92), e.g., the accumulation and over-activation of neutrophils lead to the overproduction of mucus from the cupped cells, which in turn triggers an inflammatory response; fibroblasts secrete connective tissue growth factor, leading to emphysema; and CD8^+^ cells are associated with extracellular matrix degradation and subsequent tissue remodelling associated with emphysema (93–95). Therefore, we can detect the relevant inflammatory factors and understand the pathophysiological mechanisms behind COPD through their signaling pathways, as well as improve the current therapeutic approaches and guide the further development of future research.

### Internal authenticity of included studies (limitations)

In this systematic review, there were confounders, particularly in terms of blinding and allocation concealment, which may have led to a high risk of various biases and affected the internal validity of the animal studies. Of the 453 studies, 451 (99.6%) studies were randomized controlled trials and 447 (98.7%) studies included sample size calculations, but only 109 (24.1%) studies included statements about potential conflicts of interest and research funding and 87 (19.2) studies included reasons for excluding animals from the analysis. None of the trials assessed blinding of the results, which could have led to bias in the results. Only 7.3% met four of the STAIR lists, and there were no studies that met five or more regulations. Future studies should strictly adhere to the STAIR standards for the design and implementation of animal studies and ensure full and detailed reporting of the experimental process to improve the accuracy and repeatability of animal study results. Studies should also increase the transparency of the animal study process and facilitate the translation and application of experimental results to clinics.

We initially intended to explore smoking as a contributing factor in COPD. However, as noted, there are several additional models of COPD, including those involving enhanced MMP or IL-13 expression, repetitive viral infections, chemical models with vascular endothelial growth factor (VEGF) inhibitors, and NO_2_ exposure models (96–99). We focused on the C57/B6 background, specifically of the studies using IL-38 gene knockout mice, but we observed no significant difference between the knockout and wild-type mice.

Most of the studies reviewed used only male mice to establish the COPD model to exclude the influence of estrogen on the selection of animals. This may not accurately reflect the relationship between the method of establishing the COPD model and sex. Therefore, we suggest that, when designing experiments, an equal number of male and female mice be used and that the effects of sex on the process of establishing COPD models be studied.

The measurement of lung function requires a large number of animals, specialized machines, and training, which should be addressed in future research.

## Conclusion

Smoking combined with LPS is currently the most used approach for establishing COPD animal models in a relatively short period of time, particularly with rodents. The pathogenesis of rodents is similar to that of humans; however, using rodents is more cost-effective. Although animal models of COPD cannot be directly translated into human COPD, the animal models can provide platforms for further investigation of the underlying mechanism of human COPD.

## Data Availability

The original contributions presented in the study are included in the article/supplementary material, further inquiries can be directed to the corresponding authors.
